# Supplementary material for: HIV-1 infection enhances innate function and TLR7 expression in female plasmacytoid dendritic cells
Source: Life Sci Alliance. 2022 Sep 2;5(10):e202201452. doi: 10.26508/lsa.202201452 (PMC9441429; doi:10.26508/lsa.202201452)
Supplement: Supplementary file 4 [file LSA-2022-01452_TableS1.docx]

**Supplementary Table S1**

**Table S1: PCR Primer pairs used for PCR1 and PCR2 in Fig S2A and in Fig 2A**

| Primer pair | Sequences 5’-3’ | Amplimer size |
| --- | --- | --- |
| rs3853839, pre-KASP | ACTCAGTCAGCTTCTTAAC  GGATACAGTACTTTGCAGT | 303 pb |
| rs3853839, real-time PCR | TCAGTCAGCTTCTTAACCA  CTATTTGTAGGTGGACCAT | 200 pb |
| rs179008, pre-KASP | CTTGGCACCTCTCATGCTCT  CTGTGCAGTCCACGATCACA | 225 pb |
| rs179008, real-time PCR | CTGCTCTCTTCAACCAGACCT  AAACCATCTAGCCCCAAGGAG | 140 pb |
